# B‐cell receptors of EBV‐negative Burkitt lymphoma bind modified isoforms of autoantigens

**DOI:** 10.1002/jha2.475

**Published:** 2022-06-16

**Authors:** Theresa Bock, Moritz Bewarder, Onur Cetin, Natalie Fadle, Evi Regitz, Eva C. Schwarz, Jana Held, Sophie Roth, Stefan Lohse, Thorsten Pfuhl, Rabea Wagener, Sigrun Smola, Sören L. Becker, Rainer Maria Bohle, Lorenz Trümper, Reiner Siebert, Martin‐Leo Hansmann, Michael Pfreundschuh, Hans G. Drexler, Markus Hoth, Boris Kubuschok, Klaus Roemer, Klaus‐Dieter Preuss, Sylvia Hartmann, Lorenz Thurner

**Affiliations:** ^1^ Department of Internal Medicine I and José Carreras Center for Immuno‐ and Gene Therapy Saarland University Medical School Homburg/Saar Germany; ^2^ Center for Integrative Physiology and Molecular Medicine (CIPMM) School of Medicine Homburg Germany; ^3^ Institute of Tropical Medicine Eberhard Karls Universität Tübingen Tübingen Germany; ^4^ Institute of Medical Microbiology and Hygiene Saarland University Homburg/Saar Germany; ^5^ Institute of Virology University of Saarland Homburg Germany; ^6^ Institute of Human Genetics Ulm University and Ulm University Medical Center Ulm Germany; ^7^ Helmholtz Institute for Pharmaceutical Research Saarland (HIPS) Saarbrücken Germany; ^8^ Institute of Pathology Saarland University Medical School Homburg/Saar Germany; ^9^ Department of Hematology and Oncology Georg August University Göttingen Göttingen Germany; ^10^ Dr. Senckenberg Institute of Pathology Goethe University Hospital of Frankfurt a. Main Frankfurt a. Main Germany; ^11^ Faculty of Life sciences Technical University of Braunschweig Braunschweig Germany; ^12^ Department of Internal Medicine II Augsburg University Medical Center Augsburg Germany

**Keywords:** atypical post‐translationally modified isoforms, autoantigens, BCR, Burkitt lymphoma, immunotoxins, neoantigens

## Abstract

Burkitt lymphoma (BL) represents the most aggressive B‐cell‐lymphoma. Beside the hallmark of *IG‐MYC*‐translocation, surface B‐cell receptor (BCR) is expressed, and mutations in the BCR pathway are frequent. Coincidental infections in endemic BL, and specific extra‐nodal sites suggest antigenic triggers. To explore this hypothesis, BCRs of BL cell lines and cases were screened for reactivities against a panel of bacterial lysates, lysates of *Plasmodium falciparum*, a custom‐made virome array and against self‐antigens, including post‐translationally modified antigens.

An atypically modified, SUMOylated isoform of Bystin, that is, SUMO1‐BYSL was identified as the antigen of the BCR of cell line CA46. SUMO1‐BYSL was exclusively expressed in CA46 cells with K139 as site of the SUMOylation. Secondly, an atypically acetylated isoform of HSP40 was identified as the antigen of the BCR of cell line BL41. K104 and K179 were the sites of immunogenic acetylation, and the acetylated HSP40 isoform was solely present in BL41 cells. Functionally, addition of SUMO1‐BYSL and acetylated HSP40 induced BCR pathway activation in CA46 and BL41 cells, respectively. Accordingly, SUMO1‐BYSL‐ETA’ immunotoxin, produced by a two‐step intein‐based conjugation, led to the specific killing of CA46 cells. Autoantibodies directed against SUMO1‐BYSL were found in 3 of 14 (21.4%), and autoantibodies against acetylated HSP40 in 1/14(7.1%) patients with sporadic Burkitt‐lymphoma. No reactivities against antigens of the infectious agent spectrum could be observed.

These results indicate a pathogenic role of autoreactivity evoked by immunogenic post‐translational modifications in a subgroup of sporadic BL including two EBV‐negative BL cell lines.

## 1 INTRODUCTION

Burkitt lymphoma (BL) is the most aggressive neoplasm derived from mature B‐cells. BL have been divided into endemic, sporadic and immunosuppression‐associated forms, each with different clinical manifestations and different biological characteristics. Endemic BL often manifests in childhood, localizes at the mandible and salivary glands and is suspected to be associated with EBV and *Plasmodium* infections ‐ geographically associated mainly with sub‐Saharan Africa and other tropical regions [1, 2]. Further typical sites of manifestations are the breasts of lactating women, where IgA primed B‐cells are suspected as the cells of origin [3]. Immunosuppression‐associated BL occurs in patients with variably severe T‐cell defects, such as HIV, or iatrogenically in immunosuppressed individuals following to solid organ transplantation or autoimmune disease and is like post‐transplantation lymphoma disease, usually EBV positive [1]. Sporadic BL manifests itself frequently extra‐nodally and for so far unknown reasons at the vermiform appendix/ileocecal junction and with abdominal bulks. Classic BL with *MYC*‐involving translocations t(8;14) [4], t(2;8) or t(8;22) show a similar clinical course compared to Burkitt‐like‐lymphoma with aberrant 11q [5], but the latter are increasingly regarded as a distinct entity. Expression of surface immunoglobulin (sIg) is a hallmark of all types of BL. B cell receptor (BCR)‐pathway stimulation has been reported in BL, which is characterized by mutations in *ID3* and *TCF3* genes [6] and exhibits a tonic instead of activated BCR signaling pattern [7]. CRISPR‐screening identified CD79b‐dependency in the cell line RAMOS [8]. In endemic BL, the coincidence of Malaria and EBV led to speculations, beyond the role of EBV, about a potential triggering antigen of infectious origin [9, 10]. Similar speculations exist for sporadic BL, because of the frequent initial manifestation in appendix, ileocoeliac area.

The hypothesis that activated BCR signaling by chronic antigen stimulation might contribute to the malignant transformation of B cells is old [11]. For BCRs of several B‐cell neoplasms including chronic lymphocytic leukemia (CLL), low‐grade lymphoma and multiple myeloma target autoantigens have been reported [12, 13, 14, 15, 16]. Interestingly, autoantigens in plasma cell dyscrasia were post‐translationally modified, resulting in immunogenic isoforms [17, 18, 19]. For marginal zone lymphoma, several antigens of chronic infections have been identified [20, 21, 22].

RpoC of the Gram‐negative bacterium *Moraxella catarrhalis* was recently reported by us as an antigen in IgD^+^ nodular lymphocyte predominant Hodgkin lymphoma, with an additive stimulation of LP‐cells by *Moraxella* superantigen MID/Hag via the Fc domain of sIgD [23]. Regarding more aggressive lymphomas, LRPAP1 was found to be a frequent antigen of the BCRs on mantle cell lymphoma (MCL) including the cell lines MAVER1 and Z138. In a retrospective analysis of the younger and elderly European MCL‐trials, the presence of LRPAP1‐autoantibodies was a surrogate marker for superior outcome under immunochemotherapy [24, 25]. For primary central nervous system lymphoma (PCNSL), which is a specific extra‐nodal subtype of DLBCL with molecular similarities to MCD type or C5 with frequent mutations in *MYD88* and *CD79B* [26, 27], SAMD14/neurabin‐I were recently identified by us as an antigen of the BCRs. Both were atypically post‐translationally modified (N‐hyperglycosylated) in brain tissue and PBMCs, exclusively in patients with the corresponding lymphoma BCR‐reactivity [28]. In systemic DLBCL, a cis and trans stimulation by an autoantigen was reported for cell line HBL1. The BCR (VH3‐48) of the TMD8 cells bound to an idiotope in the IgH FR2 region of its BCR [29]. Moreover, apoptotic cell debris was reported as target of the BCRs of the cell line U2932 and OCI‐LY10 [29]. Recently, we identified Ars2 as frequent antigen of BCRs of DLBCL in patients as well as in the cell lines U2932, OCI‐Ly3 and OCI‐Ly10. Ars2 was present as a hypophosphorylated isoform exclusively in Ars2‐reactive lymphoma BCRs [30].

This prompted us to screen for, and characterize, possible target antigens of BCRs of BL using expression cloning of primary cryo‐preserved specimens and BL lines, and subsequently screen for infectious antigens and post‐translationally modified autoantigens [13, 31, 32].

## 2 METHODS

The study had been approved by the local ethics committee (Ärztekammer des Saarlandes 12/13). For expression cloning of BL‐BCRs snap‐frozen specimens of patients were provided by the University Cancer Center Frankfurt (UCT). This study was approved by the institutional review boards of the UCT and the Ethics Committee (SHN‐03‐2018). Sera of a second cohort of patients with BL were obtained from Saarland University Medical Center.

BL31, BL41, CA46 and RAMOS derived from EBV‐negative sBL, DAUDI and RAJI derived of EBV‐positive eBL, and BJAB whose cell of origin is debated, were verified by their V genes. In addition, the cell lines CA46 and BL41 were ordered a second time freshly from the DSMZ (Braunschweig, Germany) and the results were verified. Cell lines of DLBCL and MCL were obtained from ATCC (Manassas, Virginia 20110–2209, United States) and DSMZ (Braunschweig, Germany). All experiments were performed with mycoplasma‐free cells.

### 2.1 BCR screening for autoantigens and antigens of infectious origin

BCRs from eight BL cell lines (BL31, BL41, CA46 and RAMOS derived from EBV‐negative sBL, DAUDI and RAJI derived of EBV‐positive eBL, and BJAB were prepared by digestion with papain. Moreover, expression cloning of recombinant BCRs derived from three primary BL cryospecimen was performed (described in supplementary). These BL line–derived BCRs and the recombinantly expressed BCRs (each at a concentration of 10 µg/ml) were pooled and screened on protein macroarrays containing clones of UniPEx 1 and 2 cDNA expression libraries (Bioscience, Dublin, Ireland), as previously described [13, 32]. To search for further antigens, all recombinant BL‐derived Fabs were screened against variously post‐translationally modified UniPEx 1 and 2 protein macroarrays, including SUMO1ylation, ubiquitination, citrullination, and acetylation. SUMOylation of protein macroarrays was performed as previously described [19] and ubiquitination was performed with synchronized HeLa cell extracts [33]. For acetylation, macroarrays were incubated with 50 mM TRIS pH8, 150 mM NaCl, 5% Glycerol, 0.1 M Trichostatin A, 10 mM EDTA, 10 mM DTT, addition of 20 pg recombinant p300 and 20 µM Coenzyme A for 30 min at 37°C. The reaction was stopped by excessive washing. GST tagged p65 (RelA) served as control for correct acetylation at Lys310.

For screening for reactivity of endemic BL BCR against *Plasmodium* antigens, all BL BCRs were tested in dot‐blots against inactivated lysates of *Plasmodium falciparum* obtained from the Institute of Tropical Medicine in Tübingen, Germany.

To screen for potential reactivity of Fabs against bacterial antigens, heat‐inactivated lysates of 13 different bacterial strains or patient isolates including *M. catarrhalis, M. osloensis, M. nonliquefaciens, Streptococcus pneumoniae*, *Streptococcus pyogenes*, *Haemophilus influenzae*, *Staphylococcus aureus*, coagulase‐negative staphylococci, *Klebsiella pneumoniae*, *Escherichia coli*, *Acinetobacter radioresistens* and *Neisseria* spp. provided by the Institute of Medical Microbiology and Hygiene in Homburg/Saar, Germany were spotted onto PVDF membranes each with a dose of 10 µg.

The dot blots of bacterial lysates were blocked in 10% (w/v) non‐fat dry milk powder in TBST (TBS, 0.1% [v/v] Tween 20) at 4°C overnight, washed with TBST and incubated for 1 h with the individual Fabs (each at 10 µg/ml), followed by further washing steps and subsequent incubation for 1 h at room temperature with biotinylated goat anti‐human heavy and light chain Fab antibody (DIANOVA, 109‐065‐088) at a dilution of 1:5,000 (v/v). Subsequently the arrays and blots were incubated for 10 min at room temperature with Strep‐POX (1:5,000) in 2% (w/v) milk/TBST and binding was detected using an ECL system (Amersham Pharmacia, Freiburg, Germany).

To screen for potential reactivities against viral antigens a custom‐made array with 2206 viral genes expressed by the NAPPA‐technology of 158 viruses was analyzed for reactivities of the BL Fabs in the Virgina C. Piper Center (Arizona state university, USA). Normalized values ≥ 2 were regarded as indicative for potential hits [34, 35, 36].

### 2.2 Expression of candidate antigens and mutants

The expression clones of BYSL and HSP40 fused C‐terminally to a tag consisting of following amino acids DYKDDDDK (FLAG‐tag) in the pSFI vector were recombinantly in HEK293 cells. Additionally, C‐terminally FLAG‐tagged full length and FLAG‐tagged fragments of different length of BYSL and HSP40 were transfected by electroporation into CA46 and BL41 cell lines via pRTS vector [37].

For secondary modification, 500 µl of cell lysates were incubated for 10 min at room temperature with 10 µl anti‐FLAG‐affinity matrix and washed afterwards. Acetylation of HSP40 and SUMOylation of BYSL were performed as previously described [19, 33]. Post‐translationally modified proteins were washed, eluted by administration of FLAG peptide (100 µg/ml), and buffered in PBS. Acetylation and SUMOylation were verified by Acetylation‐ or SUMO‐specific antibodies (Assay biotech#D0018 and BZL08843).

For site‐directed mutagenesis of BYSL and HSP40 the QuickChange II Site‐Directed Mutagenesis Kit (Stratagene, La Jolla, California, US) was used. cDNA coding full length BYSL and HSP40 each C‐terminally FLAG‐tagged, and mutants of the amino acids with the highest predicted probability as SUMOylation and acetylation sites were constructed. SUMO1‐sites in BYSL were analyzed by following exchanges of a lysine with an arginine: K36R, K78R, K110R, K139R, K154R, K158R and K194R and acetylation sites in HSP40 by K41R, K104R, K155R, K179R, K254 R, K257R, K259R, K260R, K268R, K306R, K309R, K364R, K368R, K 375R, K376R, K378R, K380R, K384R, K 390R, K403, K422R, K442, K443R, K444R. All FLAG‐tagged mutants were cloned into pRTS vector, and were transfected and expressed in CA46 or BL41 [37].

### 2.3 Production of SUMO1‐BYSL/ETA’ immunotoxin

Recombinantly expressed immunotoxin consisting of BYSL was conjugated to a truncated form of *Pseudomonas* exotoxin A (ETA’) according to Nachreiner et al. [38], and was SUMOylated as described above. It did not elicit a toxic effect in the cell line CA46, as the ETA’ component contains SUMOylation sites itself and was SUMOylated itself (not shown). Therefore, we performed an intein‐based approach [39] and expressed separately the BYSL antigen component with N‐terminally HIS6‐tag, Fu, BYSL Xa, followed by INT^N^ site EFE‐MX1^N^ and the toxin component with MBP, MXC1 as INT^C^ site followed by CEFL and ETA’. Thereafter the BYSL‐antigen component was SUMOylated as described above for 30 min at room temperature by addition of SUMO‐extract and activation buffer. Subsequently, the SUMOylated BYSL‐INT^N^ component was incubated together with the ETA’‐INT^C^ component at room temperature.

### 2.4 Enzyme‐linked immunosrobent assay (ELISA) for BCR and serum reactivity against target antigens

Acetylated HSP40 and SUMOylated BYSL were confirmed as BCR antigens by ELISA as previously described [13].

### 2.5 Western blot

Lysates of BL lines or of whole blood from patients were loaded and separated by a 10% SDS‐PAGE and transferred to PVDF membranes using a transblot semidry transfer cell (Bio Rad). After blocking overnight at 4°C in PBS/ 10% nonfat dry milk, a recombinant SUMO1‐BYSL‐ or acetylated HSP40‐reactive His‐tagged Fab were incubated at a concentration of 2 µg/ml for 1 h at room temperature, followed by incubation for 1 h at room temperature with murine anti‐His antibody at 1:2,000 (Qiagen), or with HRP‐labeled anti‐mouse IgG antibody (Bio Rad). Chemiluminescence reagent (New England BioLabs) was used for immunoblot detection. Subsequently DLBCL and MCL lines were screened for the presence of the atypic isoforms of the antigens identified in BL.

### 2.6 Proliferation and BCR pathway activation assays

For the analysis of the BCR pathway activation in CA46 cells expressing BCRs with reactivity against SUMO1‐BYSL, and in BL41 cells expressing BCRs with reactivity against acetylated HSP40, 1 × 10^6^ cells were incubated with no antigen, BYSL, SUMOylated BYSL, HSP40 or acetylated HSP40, respectively, at 5 µg/ml or anti‐IgM at 1 µg/ml for 3d at 37°C [12]. Rabbit antibodies against pTyr525/526 SYK diluted 1:2000, pTyr759 PLCγ2 diluted 1:1000, pTyr223 BTK diluted 1:1000 and pTyr96 BLNK diluted 1:1000 (B‐cell signaling sampler kit, 9768, CST, Massachusetts, USA), rabbit antibody against actin diluted 1:2000 (A5060, Sigma) and murine antibody against MYC at a concentration of 1 µg/ml, were used, followed by POX‐conjugated anti‐ rabbit or anti‐mouse antibodies diluted at 1:3000. For the analysis of proliferation or cytotoxicity, a non‐radioactive assay (EZ4U, BI 500, Biozol) was employed according to the manufacturer's instructions. Human BL lines CA46, and BL41 were used. In short, 4 × 10^4^/ml CA46 or BL41 cells were seeded in a 200‐µl cell culture medium. Recombinantly expressed BYSL, SUMOylated BYSL, HSP40 and acetylated HSP40 were added at the concentration of 1 µg/ml or the Intein‐based BYSL/ETA’ immunoconjugate [12, 24]. After 24h of incubation at 37°C, 20 µl of chromophore substrate were added to each well and adsorbance of formazan was measured at optical density (OD) 450 nm with a Wallac Victor [2].

## 3 RESULTS

Recombinant BCRs in the format of Fabs were successfully synthesized from 3 sBL cases. Moreover, antigen‐binding fragments of ‘natural’ BCRs were obtained by papain digestion from eight sporadic and three endemic BL lines. From eight of these cell lines, recombinant Fabs were generated (Table 1). Sera or plasma samples of patients with sporadic BL were obtained from the Department of Internal Medicine I of the Saarland Medical School (Homburg, Germany).

**TABLE 1:** IGV‐genes characteristics

	VH/VL segment	Homology	D segment	JH/JL segment	Homology	CDR 3 length	
Case	Gen	(%)	Gen	Gen	(%)	(AA)	Junction
BJAB	IGHV3‐30*02	91	IGHD5‐24*01	IGHJ4*02	85.4	16	CAKEGTGDGYNHYDYW
	IGKV1‐39*01	95.7		IGKJ1*01	89.5	11	CQQSYSTPWTF
BL41	IGHV4‐34*01	94.4	IGHD5‐12*01	IGHJ4*02	86.5	14	CAKAMSGSDSLNYW
	IGKV2‐28*01	99.3		IGKJ4*01	92.1	11	CMQTLQTPLTF
CA46	IGHV5‐51*01	99.3	IGHD3‐22*01	IGHJ4*02	79.2	13	CARARFDRGGDYW
	IGKV3‐20*01	99.3		IGKJ1*01	89.5	12	CQQYGSSPPWTF
Daudi	IGHV3‐74*01	83	IGHD5‐24*01	IGHJ4*02	81.3	13	CVRGNGQKCFDYW
	IGKV1‐39*01	90		IGKJ4*01	89.5	10	CQQNYNFSFTF
LAZ221	IGHV3‐23*01	98.6	IGHD1‐1*01	IGHJ4*02	81.3	15	CAKDRDWNDPSFDYW
	IGKV3‐15*01	98.2		IGKJ1*01	83.8	11	CQQYNNWPKTF
Mutu	IGHV3‐48*03	91.3	IGHD3‐3*01	IGHJ4*02	83.3	18	CARVSATSDSFLNFFDLW
	IGKV1‐27*01	95		IGKJ4*01	88.9	11	CQKYNSAPLTF
Nalm6	IGHV1‐69*12	100	IGHD6‐13*01	IGHJ6*03	100	23	CARDRRGEWPPSDYYYYYYMDVW
	IGKV1‐39*01	100		IGKJ3*01	74.3	10	CQQSYSTPWG
Raji	IGHV3‐21*06	88.2	IGHD3‐22*01	IGHJ4*02	81.3	21	CARQKNDFSDNNSYYSNFDFW
	IGKV3‐20*01	90.8		IGKJ2*01	92.3	12	CQQYGSSTLFTF
I	IGHV1‐8*01	93.8	IGHD7‐27*01	IGHJ4*02	75	12	CARAKRGVVPYW
	IGKV3‐20*01	97.9		IGKJ1*01	89.5	11	CQQYGTSPWTF
II	IGHV4‐34*01	93.7	IGHD2‐2*01	JGHJ6*02	80.7	15	CAGGNSTSYYGVDVW
	IGKV1‐17*01	96.1		IGKJ1*01	86.8	11	CLQHNNYPWTF
III	IGHV3‐30*02	97.9	IGHD6‐13*01	IGHJ6*02	75.8	20	CVRDPQQLVRGHQYCGLDVW
	IGKV3‐20*01	98.6		IGKJ1*01	92.1	11	CQQYGSSPWTF

Abbreviations: IGV, Immunglobulin variable gene; VH/VL, immunoglobulin heavy chain varriable region gene/immunoglobulin light chain varriable region gene; JH/JL, immunglobulin JH gene/immunglobulin JL gene.

### 3.1 Screening of protein macro arrays and an infective agents library

The screening against lysate of *P. falciparum* and against lysates of bacterial strains did not reveal any specific reactivity, and the screening of the BCRs against 2206 viral proteins of 158 selected viruses did not result in potential hits. In contrast, while the screening of BL Fabs on non‐modified UNIPEX1 and 2 arrays could not identify any antigenic target, screening of the same Fabs on post‐translationally modified protein macro arrays revealed SUMOylated BYSL (RZPDp829J0842 from Unipex2; UnigeneID: Hs.106880) and acetylated HSP40 (RZPDp9028H0879D from Unipex2; UnigeneID: Hs.515210) as candidate antigens.

### 3.2 Confirmation of SUMOylated BYSL and acetylated HSP40 as targets of BL‐BCRs

**FIGURE 1:**
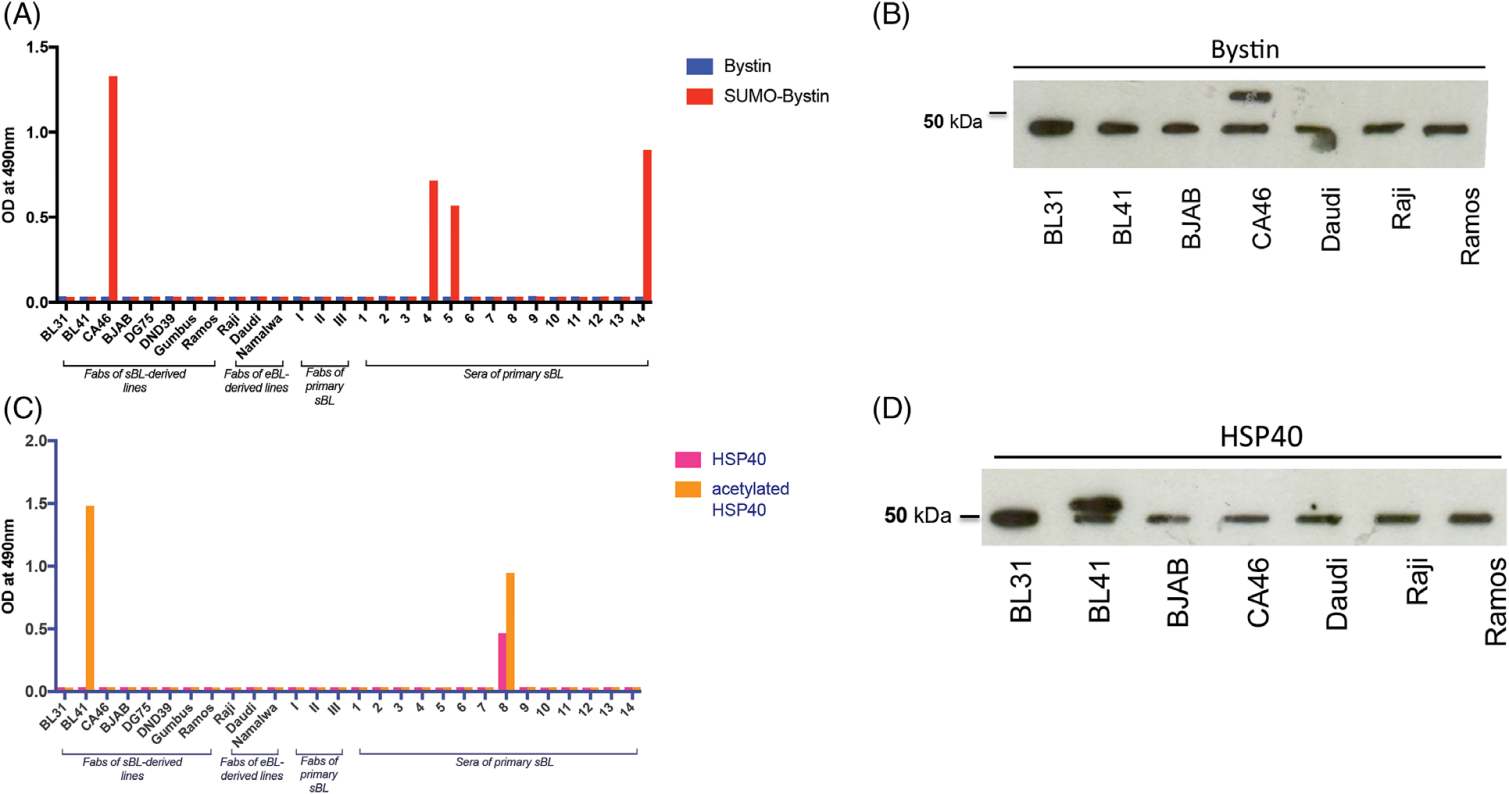
Reactivity of sporadic Burkitt lymphoma (BL) derived B‐cell receptors (BCRs) against modified autoantigens. (A) Enzyme‐linked immunosrobent assay (ELISA) for reactivity against Bystin and SUMOylated Bystin of BCRs in the Fab format derived of cell lines of sporadic or endemic BL, of cyrospecimens and of sera. The columns represent adsorbance at optical density (OD) 490 nm. (B) Western‐blot (12% SDS) of Bystin in lysates of different BL lines. A second band with a higher molecular weight was only present in the cell line CA46. (C) ELISA for reactivity against HSP40 and acetylated HSP40. (D) Western‐blot (15% SDS) of HSP40 in lysates of different BL lines. A second band with a slightly higher molecular weight was only present in the cell line BL41

ELISA with recombinant BYSL and SUMOylated BYSL or HSP40, and acetylated HSP40 expressed with a C‐terminal FLAG‐tag in HEK293, confirmed SUMOylated BYSL and acetylated HSP40 as target antigens of BL‐derived Fabs (Figure 1) and antibodies from sera of patients with BL. Fabs did not bind to unmodified BYSL and HSP40. SUMO1‐BYSL‐reactive antibodies were detected by ELISA in the sera of 3/14 (21.5%) patients with sporadic BL, with titers ranging from 1:800 to 1:3200. Acetylated HSP40 antibodies were detected in 1/14 patients (7.1%) with a titer of 1:1600, a weak reactivity against non‐acetylated HSP40 was observed in this patient.

### 3.3 Characterization of HSP40 and BYSL in BL

**FIGURE 2:**
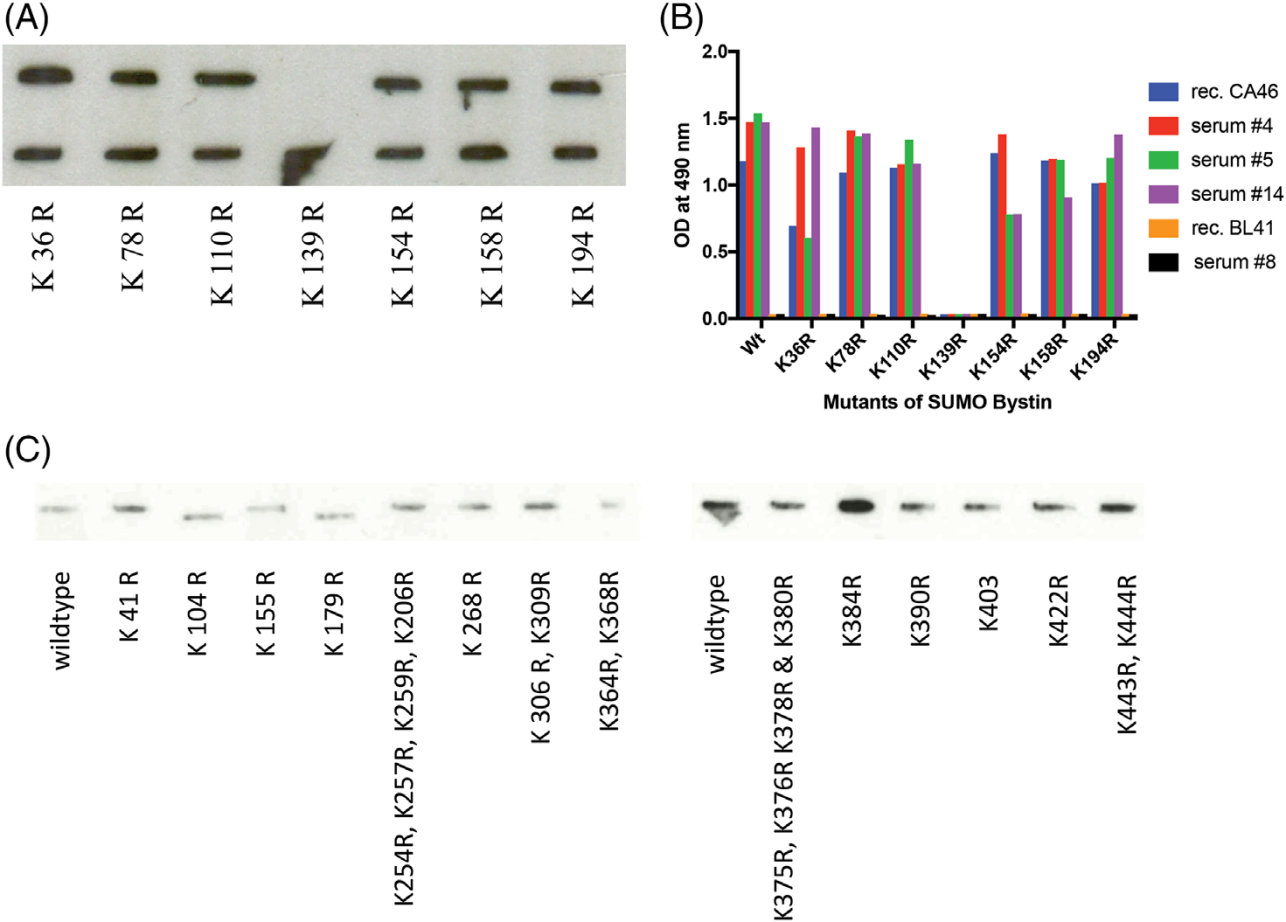
SUMOylation site of Bystin and acetylation sites HSP40 in Burkitt lymphoma (BL). (A) Western‐blot of C‐terminally FLAG‐tagged site‐directed mutants of Bystin expressed in CA46 line and identification of the SUMOylation site as amino acid 139. Murine anti‐FLAG‐antibody was used as primary antibody. (B) Enzyme‐linked immunosrobent assay (ELISA) for reactivity of Burkitt lymphoma‐B‐cell receptor (BL‐BCRs) and sera against site‐directed mutants of SUMOylated Bystin. The SUMOylated‐Bystin‐reactive BCRs and antibodies lost reactivity against K139R Bystin, which was no longer SUMOylated (see A). The columns represent adsorbance at optical density (OD) 490 nm. (C) Western‐blots of C‐terminally FLAG‐tagged site‐directed mutants of HSP40 expressed in BL41 line and identification of the acetylation sites as amino acids 104 and 179. Murine anti‐FLAG‐antibody was used as primary antibody

In Western‐blots of lysates of BL31, BL41, BJAB, CA46, Daudi, Raji and Ramos Burkitt cell lines, SUMOylated BYSL was exclusively detected in CA46 cells and acetylated HSP40 exclusively in BL41 cells (Figure 2). K139R mutagenesis of BYSL resulted in the disappearance of the SUMOylation of BYSL in CA46 cells, identifying K139 as the SUMOylation site (Figure 2). The SUMO1‐BYSL‐reactive Fabs and antibodies no longer bind to this K139R mutant of BYSL. Regarding HSP40 cells, K104 and K179 were identified as acetylation sites (Figure 2). Interestingly, Western blot analyses showed that the SUMOylated isoform of BYSL was specifically and exclusively expressed in CA46 cells with matching BCR‐reactivity, and the acetylated isoform of HSP40 was specifically and exclusively expressed in BL41 cells, with a matching BCR‐reactivity against acetylated HSP40. They were not expressed in the other BL cell lines indicated above, and not in DLBCL cell lines: (FARAGE, HBL‐1, KARPAS‐422, OCI‐Ly3, SUDHL‐6, SUDHL‐8 TMD8 and U2932) and MCL cell lines (GRANTA‐519, JEKO‐1, MAVER‐1 and Z138) (Chi‐square with Yates correction, two‐sided; *p *= 0.0341)

### 3.4 Effects of (SUMOylated) BYSL and (acetylated) HSP40 on BL cell lines, and of a SUMO1‐BYSL/ETA’ conjugate

**FIGURE 3:**
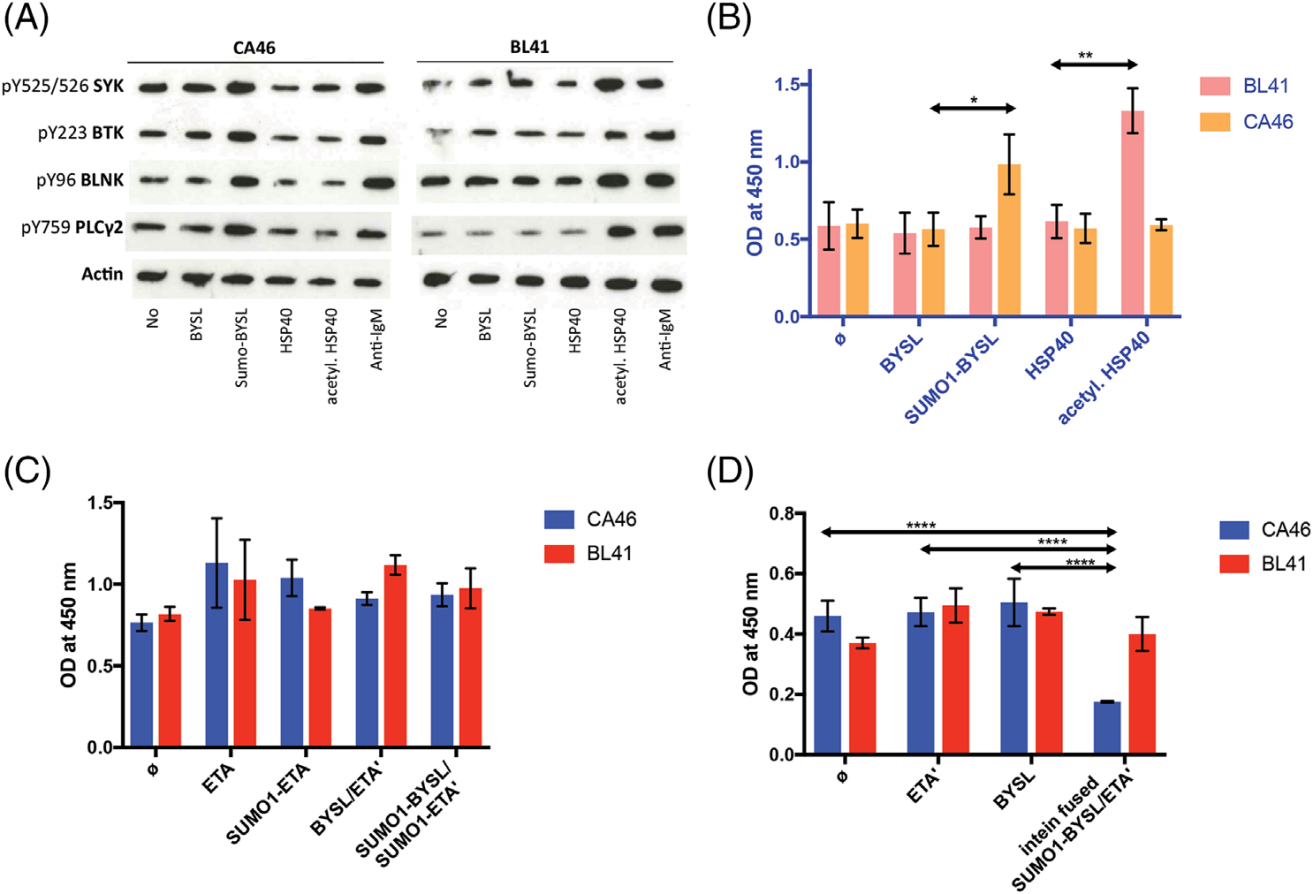
B‐cell receptor (BCR) pathway activation and induction of proliferation by SUMO1‐BYSL and acetylated HSP40 and targeting of CA46 with SUMO1‐BYSL‐containing immunotoxins. (A) Left: BCR pathway analysis by Western blot in CA46 cells upon addition of cognate antigen in the non‐ or post‐translationally modified isoform and control antigens, showed activation due to SUMOylated bystin with upregulation of pTyr525/526 SYK, pTyr96 BLNK, pTyr759 PLCγ2 and pTyr223 BTK. Right: BCR pathway analysis in BL41 cells after addition of cognate antigen HSP40 in the non‐modified or acetylated isoform and control antigens, an activation due to acetylated HSP40 was observed. (B) Induction of significant proliferation of CA46 line by SUMOylated bystin and of BL41 line by addition of acetylated HSP40. Increase of proliferation determined in the EZ4U assay (columns represent formazan at optical density (OD) of 450 nm). (C) Growth inhibition by BCR‐antigen/immunotoxins. Growth of CA46 and BL41 cells was not inhibited by addition of recombinant Bystin/ETA’, and not by Bystin/ETA’, which had been SUMOylated as a fusion protein. The SUMOylation of the fusion‐protein resulted in SUMOylation of both parts bystin and the truncated form of *P. aeruginosa* exotoxin *A (not shown), altering its function*. (D) When the immunotoxin was produced in a two‐step method using inteins, the specific toxicity could successfully be delivered. Bystin was first SUMOylated individually and then using an intein‐based binding, coupled to the toxin ETA'. Proliferation determined in the EZ4U assay (columns represent formazan at optical density (OD) of 450 nm

Western blot analysis of the stimulation of the BCR pathway upon the addition of recombinant SUMO1‐BYSL and acetylated HSP40 revealed a BCR‐pathway involvement in CA46 and BL41 cells respectively, documented through the upregulation of pTyr525/526 SYK, pTyr96 BLNK, pTyr759 PLCγ2, and pTyr223 BTK. Neither non‐SUMOylated BYSL nor non‐acetylated HSP40 induced this effect (Figure 3A). Addition of recombinant SUMO1‐BYSL and of acetylated HSP40 induced proliferation of CA46 and BL41 cells as indicated by tetrazolium/formazan EZ4U assays (Figure 3B). Addition of the SUMO1‐BYSL‐SUMO1‐ETA’ did not show any toxicity (Figure 3C), in contrast the intein‐based SUMO1‐BYSL‐ETA’ resulted in the inhibition of growth of CA46 cells but not BL41 cells (Figure 3D). No toxic effect was observed after addition of ETA’, or BYSL‐ETA’.

## 4 DISCUSSION

Here, we report the identification and characterization of two antigens recognized by the BCRs of two cell lines established from sporadic BL. BL41 is a line derived from a tumour of EBV‐negative sporadic BL from an 8‐year‐old boy [40]. CA46 is a line derived from Burkitt cells from ascites of a patient with EBV‐negative BL [41]. For both cell lines, the identified antigens turned out to be atypically modified isoforms, and we provide evidence that these modifications are associated with their immunogenicity. Similar findings have been observed in non‐malignant, mainly rheumatologic autoimmunity [42, 43, 44, 45] and also in malignant lymphoproliferative disorders including plasma cell dyscresias [17, 18, 19] as well as subsets of aggressive lymphomas such as PCNSL [28] and ABC‐type DLBCL [46].

The genesis of sporadic BL may be explained exclusively intrinsically, mainly by *IG‐MYC* rearrangements [4], mutations in *TCF3* and *ID3* [6]. Given the aggressiveness of BL, the question arises whether external stimuli such as chronic BCR stimulation by antigens play a relevant role. The results of our proliferation assays suggest that at least a subset of sporadic BL may still have an autoreactive trigger, and that this trigger may originate from atypical post‐translational modifications. In accordance with this suggestion, the antigens identified here induced BCR pathway activation and proliferation. The occurrence of SUMOylated BYSTIN, officially called Bystin like, or acetylated HSP40 exclusively in the cell line with the corresponding BCR‐reactivity is unlikely to be a coincidence. The molecular mechanisms leading to the accumulation of the atypical isoforms in these cells is unclear and should be subject to further study. In plasma cell dyscrasias, immunogenic post‐translationally modified isoforms showed an autosomal dominant inheritance pattern [15, 18, 19], which had not been observed in DLBCL or PCNSL [28, 46]. Whether an inherited susceptibility to develop an atypic and immunogenic autoantigen is involved in a subgroup of sporadic BL is currently unclear.

Therapeutically, cause and prevention of these atypical isoforms might be of interest for secondary prophylaxis. Moreover, our results indicate that artificial BCR‐antigen‐immunotoxins for targeting lymphoma cells specifically through their BCR‐reactivity may become an interesting therapeutic option for the future.

We have successfully harnessed a two‐step strategy with inteins to fuse SUMOylated Bystin to a truncated recombinant form of *P. aeruginosa* exotoxin A. However, the application of these identified BCR reactivities to target subgroups of relapsed/refractory lymphoma seems unrealistic at t present because of the rarity of the disease.

Our study is limited by the small number of cases. Unfortunately, it was not possible to obtain blood samples from adult or pediatric patients with BL despite extensive efforts. It would be very interesting to screen not only for the presence of lymphoma‐associated autoantibodies against SUMO1‐Bystin and against acetylated HSP40, but also directly for the presence of the atypical isoforms in PBMCs from patients with sporadic BL. Collecting material, together with clinical data, and making it available to the scientific community for future studies will be an important task of future clinical trials on BL.

The frequent extra‐nodal manifestation of BL in the ileocecal junction may point to a possible infectious trigger, and it is obvious that the panel of bacterial lysates that was screened reflects only a small fraction of the microorganisms that are present, for instance, in the microbiome. Additionally, most of the analyzed strains are commonly found as part of the ‘normal’ oral, pharyngeal or intestinal flora in healthy individuals. Finally, with regard to post‐translationally modified self‐proteins and degradation products, our detection tools are still limited, and the two autoantigens reported here likely represent only the tip of the iceberg. Further studies are therefore needed.

## CONFLICT OF INTEREST

The authors declare that there is no conflict of interest that could be perceived as prejudicing the impartiality of the research reported.

## ETHICS STATEMENT

The study had been approved by the local ethics committee (Ã¿rztekammer des Saarlandes 12/13) and had been approved by the institutional review boards of the UCT and the Ethics Committee (SHN‐03‐2018) of University Cancer Center Frankfurt (UCT).

## AUTHOR CONTRIBUTIONS

L. Th., N. F., E. R., K. D. P. designed the study. S H and M. L. H. performed microdissection of BL cases. R. M. B., J. H., H. D., S. R., S. L. B., S. L., B. K., S. S., J. R., T. P., R. W., L. Tr. and H. D. were of great help in the acquisition of B. L. samples and clinical data. R. S., E. S., K. R., H. D. and M. H. helped in data interpretation. N. F., T. B. and E. R. performed the protein array experiments and proteomic experiments. E. R. and T. B. performed expression cloning of Fabs and site directed point mutagenesis of HSP40 and Bystin. E. R., N. F. and K. D. P. performed intein‐based two‐step‐approach of expression of SUMOylated‐Bystin‐immunotoxins. L. Th., T. B., M. B. and K. D. P. are responsible for data analysis and interpretation of results. L. Th. wrote the manuscript. K. R., M. B., H. D., S. H., S. L. B., R. M. B., E. C. S., M. H. and R. S. revised the manuscript in detail.

## Data Availability

Additional data can be requested from the corresponding author upon request.
